# A novel strain isolated from methane seeps in the Kattegat belongs to the planctomycetal species *Novipirellula methanifontis* sp. nov.

**DOI:** 10.1038/s41598-025-33410-y

**Published:** 2026-02-13

**Authors:** Myriel Staack, Tom Haufschild, Jonathan Hammer, Nicolai Kallscheuer, Gaurav Kumar, Christian Jogler

**Affiliations:** 1 Department of Microbial Interactions, Institute of Microbiology, Friedrich Schiller University, Jena, Germany; 2 Cluster of Excellence Balance of the Microverse, Friedrich Schiller University, Jena, Germany

**Keywords:** Marine bacteria, *Pirellulaceae*, Pangenome reconstruction, Schlesner strain collection, *Planctomycetia*, Genetics, Microbiology, Molecular biology

## Abstract

Here, we present the bacterial isolate SH528^T^, a planctomycetal strain from the collection of Heinz Schlesner (Kiel University), a pioneer in the research on budding bacteria. Genomic and physiological features of the novel strain are characterized along with an analysis of its phylogenetic position within the phylum *Planctomycetota*. Strain SH528^T^ was isolated from the methane seeps of the Kattegat, a strait between Denmark and Sweden. It forms salmon-pigmented, ellipsoidal cells that divide asymmetrically by polar budding, the common cell division mode in the class *Planctomycetia*. Physiological analyses of the aerobic heterotroph revealed a mesophilic and neutrophilic lifestyle. Despite the isolation from a methane-rich environment, the strain is not a methanotroph. With a length of 10.5 Mb, the strain has a relatively large genome, even for members of the phylum *Planctomycetota*, and a DNA G + C content of 54.1%. Phylogenetic analyses using five established single gene- or genome-based markers place the strain in the family *Pirellulaceae* and support its delineation from the described species of the genus *Novipirellula*. We thus propose to assign the strain to a novel species for which we introduce the name *Novipirellula methanifontis* sp. nov. The novel taxon is represented by SH528^T^ (= DSM 116128^T^ = KCTC 102012^T^) as the type strain.

## Introduction

Representatives of the phylum *Planctomycetota* are ubiquitously distributed in aquatic and terrestrial habitats in which they contribute to the global carbon and nitrogen cycles^1^. Despite representing the fourth most abundant bacterial phylum in soil ecosystems, planctomycetes have predominantly been studied in aquatic environments^2^. In marine habitats, they frequently attach to the surface of phototrophs^3–8^, where they are part of biofilm communities and contribute to the degradation of complex polysaccharides^9–12^. Some strains exhibit a biphasic lifestyle, alternating between planktonic swimmers and sessile stalked mother cells^13^. By the specialization on recalcitrant organic macromolecules as carbon and energy sources, the slow-growing planctomycetes persist alongside faster-growing competitors such as members of the *Roseobacter* clade that occupy similar ecological niches^1,14^.

A characteristic feature of phylum members is their uncommon cell biology when compared to other Gram-negative bacteria^15,16^. Their cell envelopes bear unique crateriform structures, possibly associated with the uptake of large polysaccharides^15^. Additionally noticeable is the highly enlarged periplasmic space, that can be interpreted as a bacterial stomach allowing an internal storage and digestion of complex polysaccharides^15^. Not only the cell structure differs from most bacteria, but also the cell division which predominantly occurs by an asymmetric division mechanism (“polar/lateral budding”) that seems to be independent of the otherwise universal bacterial cell division protein FtsZ^17,18^. Apart from FtsZ, some other cell division proteins are either absent or non-essential within model species in the phylum^19,20^. Peptidoglycan, that was also long thought to be absent, has been successfully extracted from planctomycetal cell walls^16^. However, genes encoding canonical peptidoglycan biosynthesis proteins are only partially detected in their often large genomes^18,21^ and not always essential^20^. Conclusions based on the automated gene annotation often remain challenging due to a high proportion of genes with unknown function and domains that are partly unique to this phylum^22^. With the discovery of the shape-shifting *Saltatorellus* clade^23^ and the identification of “*Candidatus* Uabimicrobium amorphum” and other bacteria of prey, that take up prey bacteria via a phagocytosis-like uptake mechanism^24,25^, members of the phylum *Planctomycetota* challenge the traditional view of bacterial cell biology.

Ecological interactions are mediated by the production of small molecules^26^, which are produced by hitherto uncharacterized pathways. While the required genes are often organized as polycistronic operons in biosynthetic gene clusters (BGCs), they are scattered in planctomycetal genomes^18^. Several members of this phylum have been established as producers of diverse secondary metabolites^27–29^. Recently identified small molecules produced by *Planctomycetota* include stieleriacines (*N*-acylated tyrosine derivatives) acting as putative biosurfactants^30–32^, carotenoid pigments^33,34^, an aromatic plant toxin^35^ and alkylresorcinols of yet unknown function^36^ .

To expand the open collection of axenic cultures and gain deeper insights into the ecological role, cell biology and biotechnological potential of members of this phylum, we previously isolated and characterized more than one hundred strains from both marine and limnic environments^18,37^. Already explored sampling sites span diverse environments, including kelp forests in Monterey Bay (USA) and the North Sea (Germany)^38,39^, the macroalga *Fucus spiralis*^40^, algae in hydrothermal environments^41^, Mediterranean *Posidonia* seagrass meadows^10^, hydrothermal vents^41–43^, marine active volcanic sites^44^, plastic particles^45,46^, wood specimens^47^, marine microbial mats^48,49^, limnic cyanobacterial blooms^50^, jellyfish^51^, marine^52^ and limnic sponges^53^, sediments of seawater fishtanks^54^, wastewater^55^ and subsurface water^56^.

Here, we characterize the isolate SH528^T^, a strain from the only partly explored collection from Heinz Schlesner (a microbiologist from Kiel University). The collection was established in the 1980s to early 2000s and contains a wealth of strains belonging to uncharacterized taxa not limited to the phylum *Planctomycetota*. The here investigated strain is the first planctomycetal isolate from the methane seeps of the Kattegat, a strait between Denmark and Sweden in Northern Europe. These shallow, carbonate-cemented “bubbling reefs” recurrently release methane at rates up to 25 L/h, where aerobic methane oxidation occurs in the upper sediment layers, indicating the presence of oxygen^57^.

## Materials and methods

### Sampling, strain isolation and growth conditions

Strain SH528^T^ was sampled from methane seeps in the Kattegat, Denmark (57.510667 °N, 10.589167 °E) prior to 1990^57^. The isolated strain was revived from the cryostock originally prepared by Heinz Schlesner and was maintained in M30PY medium^58^ at a pH of 8.0. Cultures were incubated at 24 °C, if not indicated otherwise. Pure cultures were again cryopreserved in M30PY medium supplemented with 50% (v/v) glycerol and stored at − 80 °C.

## Light microscopy

Light microscopy and image analysis were performed as previously described^55^. Briefly, cultures grown to the half-maximal optical density at 600 nm (an OD_600_ of ca. 1.1) were spotted onto a 1% (w/v) agarose cushion and allowed to dry completely. A coverslip was placed over the dried mount and sample movement was prevented by fixing the coverslip in place using a 1:1:1 mixture of vaseline, lanolin, and paraffin (VLAP). Micrographs were obtained using an inverse Nikon Ti2 microscope equipped with a Nikon Plan Apo λ 100x immersion oil objective. Image acquisition was carried out with a Nikon DS-Ri2 camera and NIS-Elements software (version 5.30). Native files were converted to TIFF files in FIJI^59^ and the subsequent analysis was performed in BacStalk^60^ applying segmentation thresholds of 25 pixels for cell size and 15 pixels for minimum cell size. In total, three replicates with 150 cells each were analyzed. The web application SuperPlotsOfData was used for data visualization^61^. Brightness and contrast were adjusted manually in FIJI for visualization purposes only.

## Determination of temperature and pH optimum for growth

The temperature optimum of the strain was determined as the degree of biomass formation represented by the amount of covered surface on agar plates at nine different temperatures (4, 10, 18, 21, 24, 28, 32, 37, 42 °C) in biological duplicates. The growth behavior was evaluated visually and was documented by photographs: no growth (-), moderate growth (+), good growth (++), very good growth (+++). To determine the pH optimum for growth, M30PY medium was buffered to pH values of 5.0, 6.0, 7.0, 7.5, 8.0, 9.0, and 10.0 using 100 mM of either 2-(*N*-morpholino)ethanesulfonic acid (MES), 3-[4-(2-hydroxyethyl)piperazin-1-yl]propane-1-sulfonic acid (HEPES), or *N*-cyclohexyl-2-amino-ethanesulfonic acid (CHES) depending on the recommended buffering range. The OD_600_ values of growing cultures were measured at the determined optimal growth temperature in biological duplicates and technical triplicates in the Microplate reader Epoch2 (BioTek).

## Isolation of genomic DNA and genome sequencing

Isolation of genomic DNA and genome sequencing were performed according to the previously published workflow^55^ including the reported modifications. Basecalling, demultiplexing and filtering of reads with a Phred quality ≥ 10 were performed using Dorado version 0.5.3 (Oxford Nanopore Technologies) with the basecalling model dna_r10.4.1_e8.2 400bps_sup@v4.3.0. For quality control of the nanopore reads NanoPlot version 1.41.0 was used^62^. *De novo* assembly of the genome was performed with Flye version 2.9.3 with the flag “--nano-hq”^64,65^. Long-read polishing with Medaka (Oxford Nanopore Technologies) was omitted. Illumina sequencing was performed by Eurofins Genomics (Ebersberg, Germany) and short-read polishing was performed as described. Genome completeness of the final assembly was assessed with BUSCO v5.5.0^66,67^, while the coding density and DNA G + C content were analyzed with CheckM v1.2.3^68^. After annotation with Prokka v1.14.6^69^, the chromosome was manually rotated to the start codon of the replication initiator protein-encoding gene *dnaA* and re-annotated with PGAP version 2025-05-06.build7983.

## Nucleotide accession numbers

The 16S rRNA gene of the novel isolate is available from GenBank under the accession number PX048708. The genome sequence can be found under the accession number CP197418.

### Phylogenetic analyses

The 16S rRNA gene sequences of strain SH528^T^ and all described members of the phylum *Planctomycetota* listed in the LPSN database (https://lpsn.dsmz.de) were extracted from the annotated genomes and aligned with ClustalW^69^. The alignment was used to calculate a 16S rRNA similarity matrix with TaxonDC^70^. A 16S rRNA gene sequence-based maximum likelihood phylogenetic tree was calculated from the same alignment with FastTree 2.1^72^ employing the GTR + CAT model and 1000 bootstrap replications. Three 16S rRNA genes from bacterial strains outside of the phylum *Planctomycetota* but part of the *Planctomycetota-Verrucomicrobiota-Chlamydiota* (PVC) superphylum, namely *Opitutus terrae* (NCBI accession number AJ229235), *Kiritimatiella glycovorans* (NCBI accession number NR_146840) and *Lentisphaera araneosa* (NCBI accession number NR_027571), served as outgroup. The multi-locus sequence analysis (MLSA)-based phylogenetic analysis was performed using autoMLST with 500 bootstrap replicates^72^. The analysis was performed with the autoMLST-simplified-wrapper tool available on GitHub based on the reference genome of all current members of the family *Pirellulaceae*. The genomes of the following three members of the family *Planctomycetaceae* were used as outgroup: *Planctopirus limnophila* DSM 3776^T^ (accession number GCF_000092105.1), *Rubinisphaera brasiliensis* DSM 5305^T^ (accession number GCA_000165715.3) and *Planctomicrobium piriforme* P3^T^ (accession number GCA_900113665.1). Average amino acid identities (AAI) and average nucleotide identities (ANI) were calculated using the respective scripts of the enveomics collection^73^. The percentage of conserved proteins (POCP) was calculated as described^74^. The *rpoB* gene sequences coding for the β-subunit of DNA-directed RNA polymerase were taken from publicly available online databases and sequence identities of the previously used 1298 bp partial sequence were determined as previously described^75,76^.

## Analysis of genome-encoded features

The number of genes coding for putative carbohydrate-active enzymes (CAZymes) was obtained from the genome annotation provided by dbCAN3^78^. An in silico prediction of BGCs putatively involved in the biosynthesis of secondary metabolites was carried out using antiSMASH 7^79^. The prediction was run with relaxed strictness and all extra features activated. The pangenome of the compared type strains of *Novipirellula* spp. was reconstructed using anvi’o v.8 with default parameters^80^.

## Results and discussion

### Phylogenetic inference

**Fig. 1:**
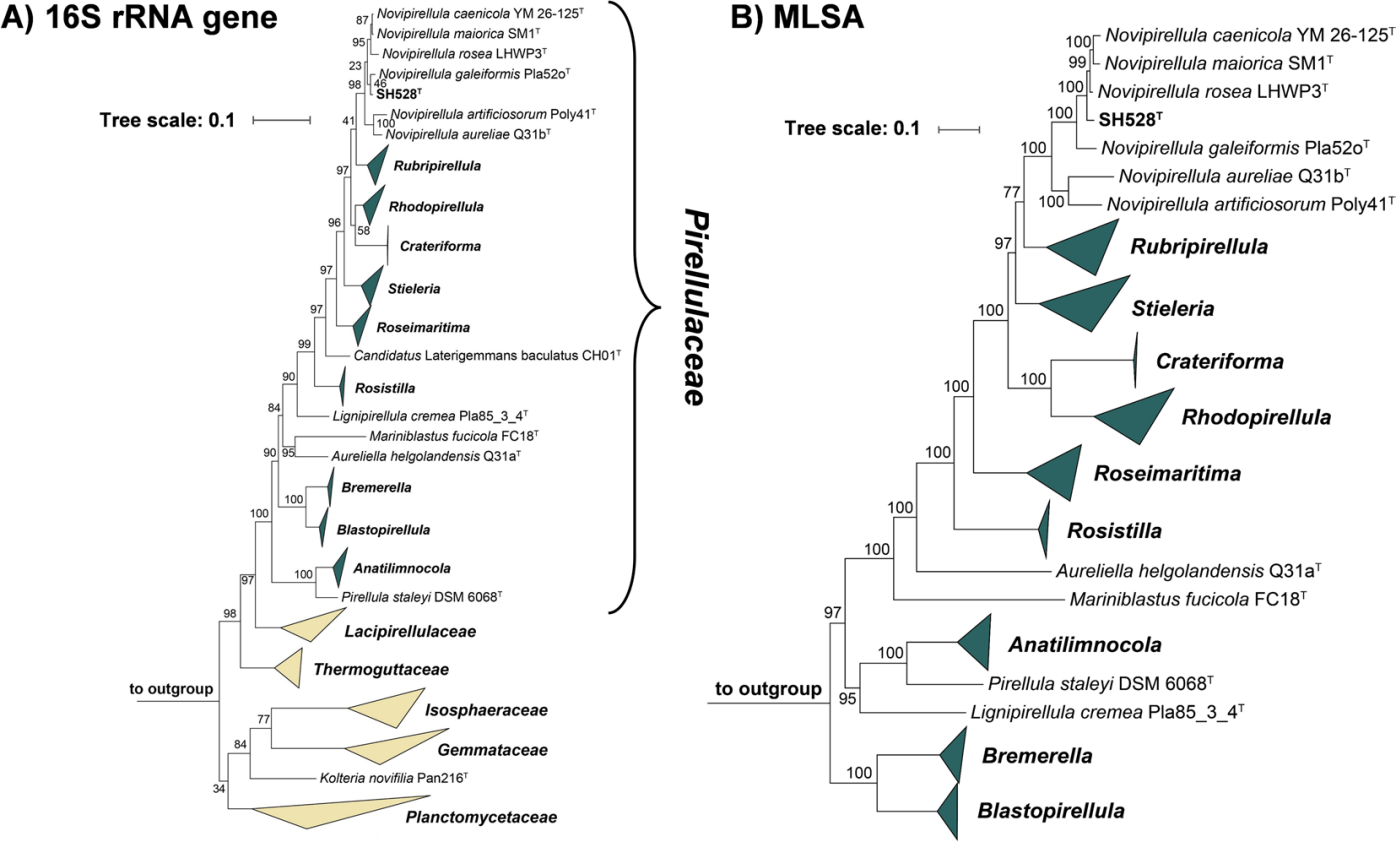
Maximum likelihood 16S rRNA gene sequence- and MLSA-based phylogenetic trees. The phylogenetic trees display the position of strain SH528^T^ in relation to their closest relatives. Both phylogenetic trees were computed using a maximum likelihood approach. Pale yellow triangles indicate collapsed families whereas dark green triangles indicate collapsed genera within the family *Pirellulaceae* . The bootstrap values after 1000 re-samplings (16S rRNA gene sequences)/ 500 re-samplings (MLSA) are provided at the nodes (in %). Sequences used as outgroups are provided in the material and methods section. The scale bar indicates the number of substitutions per nucleotide position (16S rRNA gene sequence-based tree) or amino acid position (MLSA-based tree).

**Fig. 2:**
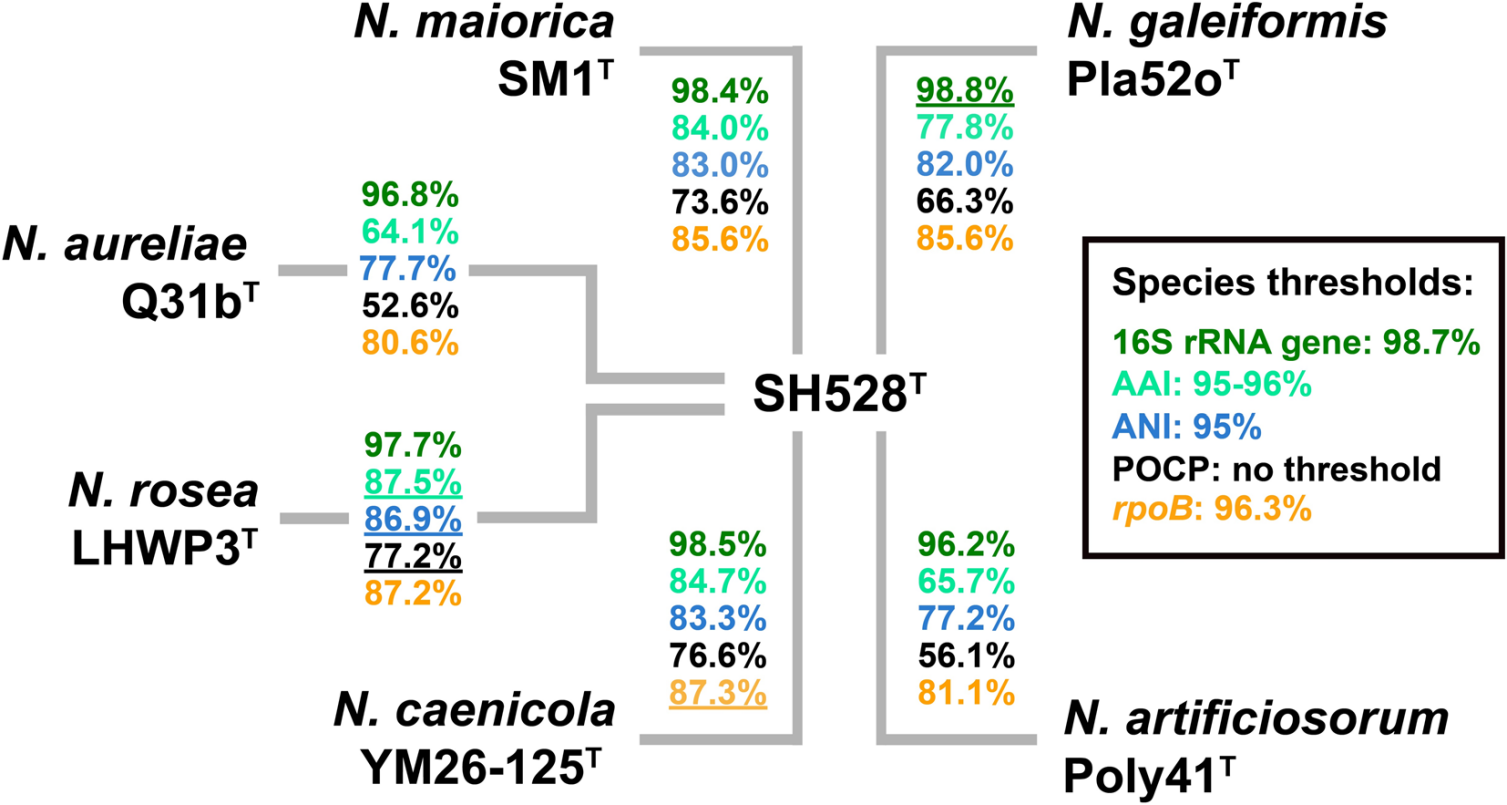
Comparison of phylogenetic markers. Analyzed markers included 16S rRNA gene sequence similarity, average amino acid identity (AAI), average nucleotide identity (ANI), percentage of conserved proteins (POCP) and *rpoB* gene sequence similarity. The highest value of each phylogenetic marker during comparison with strain SH528^T^ is underlined.

In phylogenetic trees constructed based on 16S rRNA gene sequences and MLSA, strain SH528^T^ clustered in the family *Pirellulaceae*, more specifically within the clade that includes the current members of the genus *Novipirellula*. The clustering patterns in the two trees suggested different strains as the current closest neighbors of the novel isolate: in the 16S rRNA gene sequence-based tree, strain SH528^T^ clustered next to *Novipirellula galeiformis* Pla52o^T^, whereas in the MLSA-based tree it clustered next to *Novipirellula rosea* LHWP3^T^, *Novipirellula maiorica* SM1^T^ and *Novipirellula caenicola* YM26-125^T^ (Fig. 1). The clustering of strain SH528^T^ with *N. galeiformis* Pla52o^T^ in the 16S rRNA gene sequence-based tree is in line with the finding that both strains share the highest 16S rRNA gene sequence similarity of 98.8%. However, this similarity value is slightly above the established species threshold of 98.7%^81^, indicating a relationship on the level of the same species (Fig. 2). The 16S rRNA gene sequence identities with the other three close relatives fell below the species threshold of 98.7%. As expected from the clustering pattern in the trees, the analyzed phylogenetic markers also suggested different strains as the current closest neighbor of the novel isolate (underlined values shown in Fig. 2). ANI values obtained during comparison of strain SH528^T^ with the four above-mentioned strains ranged from 77 to 87% and thus fell significantly below the species threshold of 95%^81,82^, thereby excluding that the novel isolate belongs to an existing species (Fig. 2). Values for AAI, POCP and partial *rpoB* sequence identity further support the genus-level affiliation while indicating species-level separation. Thus, the 16S rRNA gene sequence is the only marker suggesting that *N. galeiformis* Pla52o^T^ and strain SH528^T^ may belong to the same species. Such a discrepancy is not unexpected since the species threshold for the 16S rRNA gene sequence similarity is highly unreliable for members of the phylum *Planctomycetota*. In the past, strains could be assigned to separate species although their 16S rRNA genes only differed at two nucleotide positions^53^. Thus, the obtained values are in line with the conclusion that strain SH528^T^ belongs to a separate species.

### Genomic and genome-encoded features

Strain SH528^T^ represents the first described member of the genus for which a complete genome (i.e. a single circular contig) could be obtained. With 10.5 Mb and an estimated completeness of 99.8% it is also the largest genome compared to previously described relatives^76^ (Table 1). The genome sizes of the closest relatives range from 7.4 to 10.0 Mb and the available draft genome assemblies are highly fragmented (40–1132 contigs). The DNA G + C content of strain SH528^T^ of 54.1% is within the usual range for members of the genus *Novipirellula*^76^. Coding sequences make up 85.7% of the entire genome sequence. The automated genome annotation with PGAP yielded 7,298 protein-coding genes of which 25.9% (1893) are annotated as hypothetical proteins. Obtained values are consistent with those observed in other members of the genus, typically showing a coding density of approximately 85% and relative numbers of hypothetical proteins ranging from 24 to 28% (Table 1). Strain SH528^T^ harbors 101 tRNA genes, along with two copies of 5S, 16S and 23S rRNA genes each. Extrachromosomal elements are not present in the novel isolate. Information on the presence of plasmids in the close relatives remains speculative based on the incomplete draft genomes.

**Table 1:** Genomic and genome-encoded features.

Characteristics	SH528^T^	*N*. *galeiformis* Pla52o^T^	*N*.* rosea* LHWP3^T^		*N*. *caenicola* YM26-125^T^	*N*. *maiorica* SM1^T^
*Genomic features*						
Genome size (bp)	10,459,951	7,403,604	9,994,186		8,878,136	8,874,084
Contigs	1 (closed)	40	140		78	1,132
Plasmids	no	inconclusive	inconclusive		inconclusive	inconclusive
DNA G + C (%)	54.1	55.8	54.9		55.4	54.7
Completeness (%)	99.8	99.3	99.6		99.8	93.2
Genes	7,446	5,356	7,222		6,293	7,050
Genes/Mbp	712	723	723		709	794
Protein-coding genes	7,298	5,272	7,108		6183	6,660
Protein-coding genes/Mbp	698	712	711		696	750
Hypothetical proteins*	1,893	1,238	2,000		1,626	1,642
Hypothetical proteins (%)	25.9	23.5	28.1		26.3	24.7
Coding density (%)	85.7	84.7	85.9		84.6	86.4
rRNA genes (5S,16S,23S)	2,2,2	2,3,2	n.d.,3,2		3,4,2	1,1,1
tRNA genes	101	44	52		71	88
*Secondary metabolite-associated biosynthetic gene clusters*						
Terpene (incl. precursors)	3	3	3		3	4
Type I polyketide synthase	2	0	1		2	1
Mixed type I polyketide synthase-non ribosomal peptide synthetase	1	0	2		1	1
Type III polyketide synthase	1	1	1		1	1
Phosphoglycolipid	1	0	0		0	0
Ribosomally-synthesized peptide-related	0	0	0		1	0
Non-ribosomal peptide synthetase-like	2	3	2		2	2
Acyl amino acid	0	0	0		0	1
BGCs (total)	10	7	9		10	10
BGCs per Mbp	0.8	0.5	0.7		0.8	0.8
*Carbohydrate-active enzyme-encoding genes*						
Glycoside hydrolases	132	61	101		94	96
Glycosyltransferases	93	68	85		71	93
Polysaccharide lyases	11	6	8		7	7
Carbohydrate esterases	34	31	29		30	31
Carbohydrate-bind. modules	22	12	21		19	21
Auxiliary activities	4	3	5		4	5
CAZyme genes (total)	296	181	249		225	253
CAZyme genes per Mbp	28	24	25		25	29

*Based on the Refseq-annotated genomes, n.d. not detected (due to low quality of the assembly).

Genome mining with antiSMASH yielded ten BGCs in the genome of strain SH528^T^ (Table 1). These include three clusters putatively involved in terpenoid production, two coding for putative type I polyketide synthases (PKS), one for a mixed type I PKS/non-ribosomal peptide synthase (NRPS), one for a type III PKS, two for NRPS-like enzymes and one enzyme probably involved in phosphoglycolipid biosynthesis (Table 1). Strain SH528^T^ harbours nearly the same set of BGCs as *N. rosea* LHWP3^T^, *N. caenicola* YM26-125^T^ and *N. maiorica* SM1^T^, reflecting their close phylogenetic relatedness. However, some strain-specific differences were observed. Strain SH528^T^ harbors an additional putative cluster for phosphoglycolipid production, *N. caenicola* YM26-125^T^ a specific cluster related to ribosomally-synthesized peptide synthesis and *N. maiorica* SM1^T^ a cluster for acyl amino acid production. The genome of the novel isolate was also analyzed for its genomic potential to build, modify and break down complex carbohydrates. In total, 296 genes encoding putative carbohydrate-active enzymes (CAZymes) were identified in the genome (Table 2). *N. rosea* LHWP3^T^ and *N. maiorica* SM1^T^ encode a similarly high number of CAZymes with particularly high numbers of glycoside hydrolases and glycosyltransferases. In contrast, *N. galeiformis* Pla52o^T^ only harbors 181 putative CAZyme genes with only half of the amount of glycoside hydrolases compared to strain SH528^T^, consistent with its smaller genome. The presence of a high number of CAZyme genes, particularly encoding glycoside hydrolases, suggests a broad variety of carbohydrates that can be utilized, which may provide an advantage in environments with low availability of mono- or oligosaccharides. Since strain SH528^T^ was isolated from methane seeps, its genome was also examined for genes involved in methane uptake, oxidation and assimilation. Typical marker genes, such as those encoding methanol dehydrogenase or methane monooxygenase, were not detected, suggesting that the strain is not methanotrophic. Consistent with the typical physiology of the genus *Novipirellula*, strain SH528^T^ grows strictly aerobically. The genomic analysis revealed only an incomplete set of genes putatively associated with nitrate and sulfite metabolism, including *nrtD* (nitrate importer), *narB* (nitrate reductase), *napA* (periplasmic nitrate reductase), *norR* (anaerobic nitric oxide reductase transcription regulator) and *asrA* (anaerobic sulfite reductase subunit A). These genes encode either regulators or enzymes that catalyze partial steps. Since no complete set of terminal reductases required for anaerobic respiration was found, there is insufficient support for anaerobic respiration performed by strain SH528^T^.

**Table 2:** Phenotypic features.

Characteristics	SH528^T^	*N*. *rosea* LHWP3^T^	*N*. *galeiformis* Pla52o^T^	*N*. *caenicola* YM26-125^T^	*N*. *maiorica* SM1^T^
*Sampling information*					
Location	Kattegat, Denmark	Ark clam farm in Gangjin Bay, south coast of Korea	Baltic Sea at Heiligendamm Pier, Germany	Murohama Beach, Kamaishi, Iwate, Japan	Mallorca, Spain
Sampled material	Methane seeps	Dead ark clam *Scapharca broughtonii*	Pieces of wood embedded in incubator in the water of Baltic Sea (2 m depth)	Iron sand (30 cm depth from surface)	Sediment
*Phenotypic features*					
Pigmentation	Salmon-pigmented	Pink to red	Light pink	Pink	Light pink
Cell shape	Ellipsoid to round	Ovoid	Acorn, strongly deformed	Spherical	Almost round
Size (length x width) (µm)	1.4 ± 0.1 x 1.1 ± 0.1	0.6–1.5 x 0.6–1.4	2.0 ± 0.4 x 1.0 ± 0.2	1.0–1.1 (diameter)	1.2–1.6 × 1.0–1.4
Cell division mode	Polar budding	Polar budding	Polar budding	Polar budding	Polar budding
Temperature range (optimum) (°C)	10–24 (24)	20–37 (30)	10–36	20–30 (28)	16–37 (28–32)
pH range (optimum)	6.0–10.0 (8.0)	6.0–8.0 (7.0)	5.5–9.0	6.0–8.0 (7.0)	6.5–10.0 (7.5-8.0)
Relation to oxygen	Aerobic	Aerobic	Aerobic	Aerobic	Aerobic
Stalks	n.o.	n/a	n.o.	n/a	n/a
Aggregates	Yes	n/a	yes, 10–30 cells/ aggregate	yes	yes

n.o. not observed, n/a data not available. Data for the strains used for comparison was taken from the original species description articles^76,83,84^

**Fig. 3:**
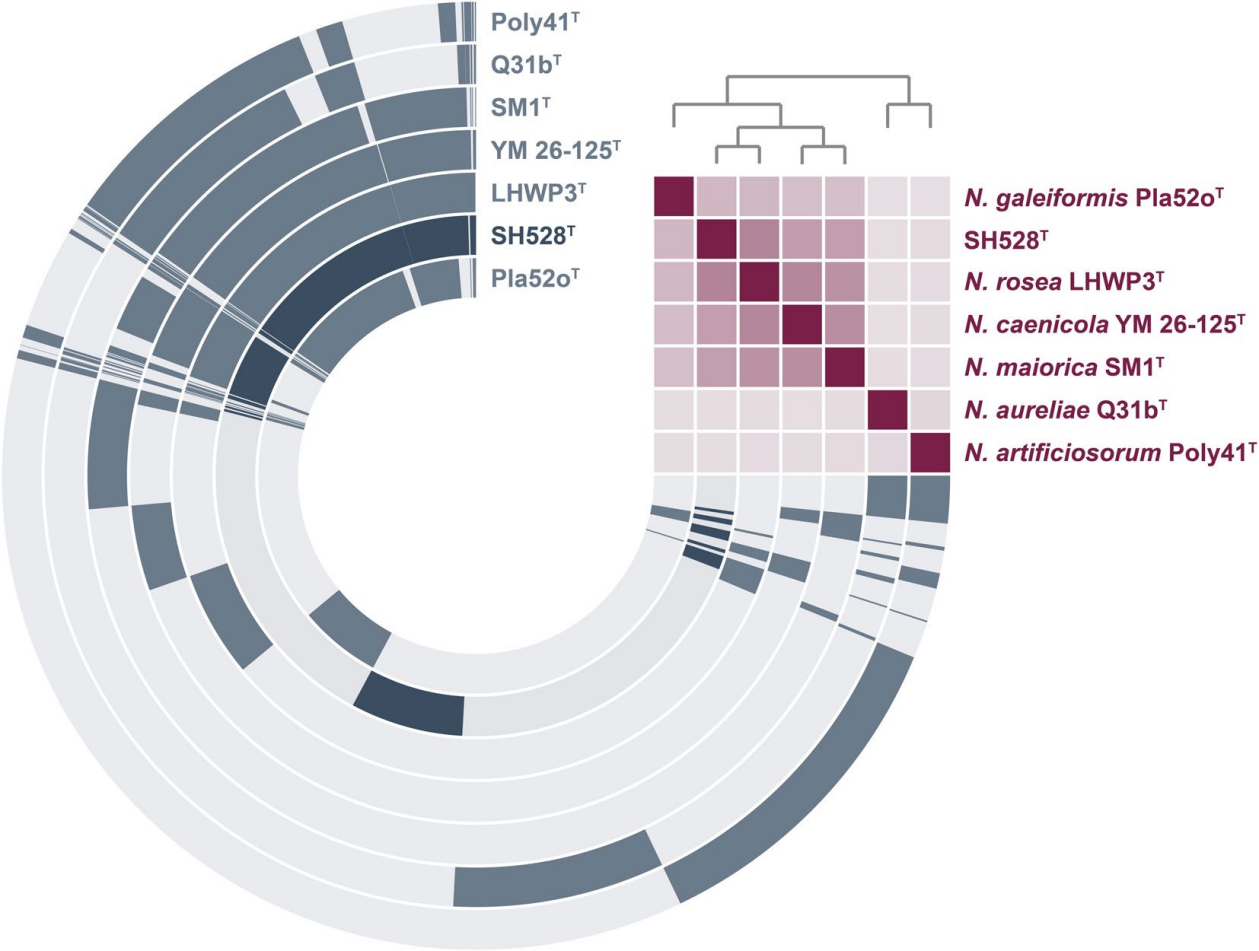
Pangenome of strain SH528^T^ and previously described *Novipirellula* species. The concentric rings represent the gene content of the analyzed genomes. Dark-colored segments indicate the presence of the gene in the respective genome, while light-colored segments represent gene segments without homologs in the other genome. The heatmap in the upper right corner illustrates the genome relatedness of the strains based on ANI values with faint purple corresponding to ≤ 70% and bright purple to 100% identity.

The reconstructed pangenome of strain SH528^T^ and the identified closely related members of the genus *Novipirellula* comprises 18,724 gene clusters with a core genome of 1,951 clusters (10.4%) conserved across all analyzed genomes (Fig. 3). The low proportion of core genes may indicate that *Novipirellula* species exhibit a high genomic diversity with many species-specific genes. Despite that, it may also be caused because they do not cluster well together due to selected thresholds, sensitivity during clustering or weak homology. The gene presence/absence visualization of the pangenome further highlights the closer relationship of SH528^T^ to *N. rosea* LHWP3^T^, *N. caenicola* YM26-125^T^ and *N. maiorica* SM1^T^ as indicated by the ANI-based heat map (upper right corner in Fig. 3). A total of 1,755 clusters are unique to strain SH528^T^, representing 9.4% of the pangenome. The presence of singletons in addition to the large set of CAZymes may reflect species-specific functional adaptations that enable strain SH528^T^ to thrive in its ecological niche.

### Characterization of morphological and physiological characteristics

**Fig. 4:**
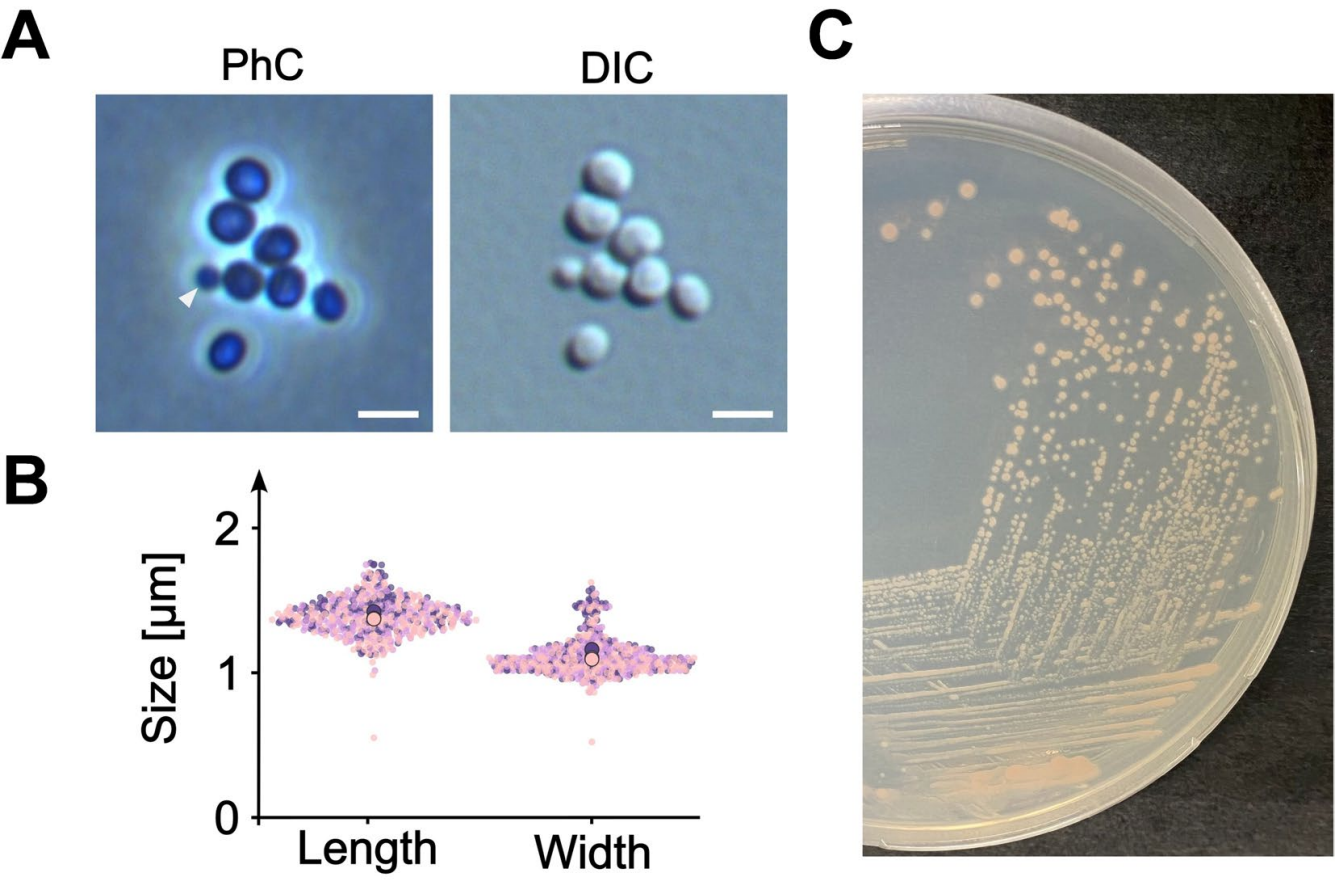
Morphological properties of the novel isolate. Phase contrast (PhC) and differential interference contrast (DIC) images highlighting cell division mode (white triangles) and cell morphology (**A**). The scale bars represent 2 μm. Three replicates, distinguished by colored dots with 150 analyzed cells each, were used to determine cell length and width (**B**). Mean values for each replicate are indicated by larger circles. On agar plates, cell form salmon-pigmented round colonies (**C**).

Microscopic images depicting individual cells of strain SH528^T^ revealed a cell shape ranging from ellipsoid to round (Fig. 4A). While mother cells often appeared round, daughter cells appeared ellipsoid to acorn-shaped instead. The latter shape is consistent with the shape of *N. galeiformis* Pla52o^T^, while other relatives like *N. caenicola* YM26-125^T^ or *N. maiorica* SM1^T^ show more roundish cells (Table 2). In liquid culture, strain SH528^T^ formed shapeless aggregates, like the other species of the genus *Novipirellula*. Cells are 1.4 ± 0.1 μm in length and 1.1 ± 0.1 μm in width (Fig. 4B). This observation renders cells of this strain shorter than those of *N. galeiformis* Pla52o^T^ with 1.9 × 1.0 μm, but longer compared to the round cells of strain *N. caenicola* YM26-125^T^. Strain SH528^T^ divides by asymmetric cell division, also referred to as polar budding, a common feature of all members of the order *Pirellulales* (and most other orders in the class *Planctomycetia*). A small round daughter cell emerges on one cell pole, increases in size and elongates over time and eventually completes the division process by pinching off from the previously larger mother cell (Fig. 4A).

Colonies of strain SH528^T^ are salmon-pigmented and have entire margins (Fig. 4C), displaying the characteristic colony pigmentation of the genus in varying shades of pink (Table 2).

Growth of the strain occurs under aerobic conditions and at temperatures between 10 and 24 °C, with an optimum at 24 °C on M30PY agar plates. In general, members of the genus *Novipirellula* cover a broad temperature range from 10 to 37 °C, in which strain SH528^T^ shows growth at the lower boundary. The strain tolerates an external pH range of 6.0–10.0, showing optimal growth at pH 8.0. The physiological properties are in line with the so far described members of the genus that are all mesophilic neutrophiles.

With the description of the novel isolate, we here expand the open collection of axenic cultures of *Novipirellula* spp. Members of the genus are not only exceptionally rich in CAZyme-encoding genes but also show potent antimicrobial activities^82^. Hence, the genus holds hidden biotechnological potential in addition to the ecological significance.

## Conclusion

Based on the phylogenetic inference and supported by differences in genomic and phenotypic features, it can be conducted that strain SH528^T^ belongs to a novel species of the genus *Novipirellula*, for which we propose the name *Novipirellula methanifontis*.

Description of *Novipirellula methanifontis* sp. nov.

*Novipirellula methanifontis* (me.tha.ni.fon’tis. N.L. neut. n. *methanum*, methane; L. gen. n. *fontis*, of a spring or source; N.L. gen. n. *methanifontis*, of a methane spring, referring to the isolation of the type strain from a methane seep).

Colonies are round and salmon-pigmented. Cells are ellipsoid to round in shape (length: 1.4 ± 0.1 μm, width: 1.1 ± 0.1 μm) and divide asymmetrically. Cells are aerobic heterotrophs. The type strain is SH528^T^ (DSM 116128^T^ = KCTC 102012^T^). It was isolated from methane seeps at the Kattegat, Denmark. The type strain grows at temperatures between 10 and 24 °C (optimum 24 °C) and tolerates external pH values from 6.0 to 10.0 (optimum 8.0). Its genome has a size of 10.5 Mb with a DNA G + C content of 54.1%. Extrachromosomal elements are not present.

## Data Availability

The 16S rRNA gene of the novel isolate is available from GenBank under the accession number PX048708. The genome sequence can be found under the accession number CP197418.
